# Using the biopsychosocial model for identifying subgroups of detained juveniles at different risk of re-offending in practice: a latent class regression analysis approach

**DOI:** 10.1186/s13034-021-00379-1

**Published:** 2021-06-22

**Authors:** E. L. de Ruigh, S. Bouwmeester, A. Popma, R. R. J. M. Vermeiren, L. van Domburgh, L. M. C. Jansen

**Affiliations:** 1 Department of Child and Adolescent Psychiatry & Psychosocial Care, Amsterdam UMC -VUmc, Meibergdreef 5, 1105 AZ Amsterdam, The Netherlands; 2 Erasmus University Rotterdam, Rotterdam, The Netherlands; 3 Department of Child and Adolescent Psychiatry, Leiden University Medical Center, Oegstgeest, The Netherlands; 4 Pluryn-Intermetzo, Lelystad, The Netherlands

**Keywords:** Juvenile offenders, Reoffending, Latent class regression, Subgroups, Neurobiology, Risk assessment

## Abstract

**Background:**

Juvenile delinquents constitute a heterogeneous group, which complicates decision-making based on risk assessment. Various psychosocial factors have been used to define clinically relevant subgroups of juvenile offenders, while neurobiological variables have not yet been integrated in this context. Moreover, translation of neurobiological group differences to individual risk assessment has proven difficult. We aimed to identify clinically relevant subgroups associated with differential youth offending outcomes, based on psychosocial and neurobiological characteristics, and to test whether the resulting model can be used for risk assessment of individual cases.

**Methods:**

A group of 223 detained juveniles from juvenile justice institutions was studied. Latent class regression analysis was used to detect subgroups associated with differential offending outcome (recidivism at 12 month follow-up). As a proof of principle, it was tested in a separate group of 76 participants whether individual cases could be assigned to the identified subgroups, using a prototype ‘tool’ for calculating class membership.

**Results:**

Three subgroups were identified: a ‘high risk—externalizing’ subgroup, a ‘medium risk—adverse environment’ subgroup, and a ‘low risk—psychopathic traits’ subgroup. Within these subgroups, both autonomic nervous system and neuroendocrinological measures added differentially to the prediction of subtypes of reoffending (no, non-violent, violent). The ‘tool’ for calculating class membership correctly assigned 92.1% of participants to a class and reoffending risk.

**Conclusions:**

The LCRA approach appears to be a useful approach to integrate neurobiological and psychosocial risk factors to identify subgroups with different re-offending risk within juvenile justice institutions. This approach may be useful in the development of a biopsychosocial assessment tool and may eventually help clinicians to assign individuals to those subgroups and subsequently tailor intervention based on their re-offending risk.

**Supplementary Information:**

The online version contains supplementary material available at 10.1186/s13034-021-00379-1.

## Introduction

Antisocial youth are known to be a heterogeneous group, for example in relation to psychiatric disorders and mental health needs (e.g. [1]). This heterogeneity bears relevance for development, course, and prognosis of antisocial behavior [2]. Research focusing on more accurate prediction of reoffending is vitally important, especially as reoffending rates of juvenile delinquents in the Netherlands have shown a smaller decline over recent years than e.g. adult offenders [3]. Although the number of juvenile delinquents in The Netherlands has decreased in the last decade, recidivism rates remain high, around 38%. Specifically, recidivism rates of juvenile delinquents after a stay in a Juvenile Justice Institution (JJI) within 3 years after release is 63%, and 17% for serious recidivism [4]. Distinguishing more homogeneous subgroups of juvenile offenders may help in better predicting and eventually preventing future delinquent behavior in clinical forensic practice. A growing body of research shows that neurobiology is increasingly seen as a valuable additional aspect to improve risk assessment and intervention allocation in clinical forensic practice [5–7]. However, biological measures are currently seldom used in clinical forensic settings. Therefore, we set out to study whether the use of relatively easy- to- measure neurophysiological and neuroendocrinological correlates of antisocial behavior could help us to identify homogenous subgroups of detained juveniles at different risk of re-offending in a sample of detained juvenile delinquents.

Previous research in clinical forensic practice has focused on defining homogeneous subgroups of juvenile delinquents, based on offender characteristics such as environmental factors, psychological and individual characteristics, personality traits, or combinations thereof [2, 8–12]. Frequently, clusters are found that vary in terms of severity, for example in terms of problem behavior (e.g. [10, 13–15]). In addition, integration of risk factors for antisocial behavior, problem behavior and mental health problems, substance use, and psychopathic traits, often prove important when clustering detained juveniles [2, 10, 12, 13]. Relevant subgroups generally differed in the number of problems in various domains, ranging from subgroups with few problems to subgroups with problems across all domains [12, 14]. In addition, specific subgroups were identified with high levels of mainly externalizing psychopathology and/or psychopathic traits [13].

Several studies have shown that identifying subgroups is relevant for identifying recidivism risk. For example, in serious juvenile offenders a subgroup of violent property offenders, who were also identified as the most serious recidivists, had the highest number of risk factors [16]. Another study showed that most chronic and severe offenders were adolescents with affective, interpersonal, as well as behavioral psychopathy dimensions present [17]. As for severity of recidivism, a distinction is made between non-violent and violent recidivism. Factors predictive of recidivism have been shown to be different for violent young offenders as compared to non-violent offenders [18]. Based on these factors, a different focus regarding (the allocation to) interventions seems warranted (see e.g. [10, 19]).

Although previous studies yielded clinically relevant clusters, they were usually based on psychosocial offender characteristics alone. However, a growing body of research shows that neurobiology is increasingly seen as a valuable additional aspect to understanding persistent antisocial behavior [20–23], as is also depicted in the biopsychosocial model [24]. Based on this knowledge, several researchers argue in favor of a biopsychosocial framework [5–7], which states that interactions between psychosocial and neurobiological factors influence the development of antisocial behavior [6, 25, 26]. Furthermore, developmental crime prevention programs that recognize both the importance of the environment and biology were shown to reduce crime [27]. So far, neurobiological measures are not commonly used in risk assessment and intervention allocation in clinical forensic practice.

Research has identified several potential peripheral biomarkers of antisocial behavior that can be relatively easy applied in clinical practice, such as heart rate (HR), cortisol, and testosterone (e.g. [28–30]). HR is an indicator of activity of both the parasympathetic (PNS) as well as the sympathetic (SNS) branch of the autonomic nervous system (ANS). Meta-analyses showed that low HR in rest and in reactivity to a stressor is a consistent biological correlate of antisocial behavior in juveniles [28, 29]. Longitudinally, low resting HR at age 15 was associated with criminal status at age 24 [31], and persistent delinquent behavior in a subsample from this population [32]. In several studies, lower resting heart rate levels were related to persistent offending behavior [29, 33, 34], while others found only HR and HRV reactivity to be related to reoffending [35, 36].

With regard to SNS functioning, poor autonomic fear conditioning at age 3 was associated with criminal offending 20 years later [37]. Furthermore, diminished SNS reactivity came forward as risk factor for externalizing problems [38]. Low cortisol and high testosterone levels (easily measured in saliva) have also been associated with antisocial behavior (e.g. [39]). Although mixed results have been found with respect to cortisol levels and antisocial behavior, a negative relationship between decreased cortisol (re)activity and antisocial behavior is reasonably consistent [40, 41]. A meta-analysis showed a positive relationship between testosterone and aggression, however not all studies find this link [42]. Growing evidence shows cortisol and testosterone are interdependent in their influence on antisocial behavior (e.g. [43]), and should therefore both be studied.

However, the use of neurobiological measures in daily practice still faces major challenges, as inferences on an individual level cannot be made based on the studies mentioned above. As Moffitt et al. [44] stated, there is no consensus on when biomarker levels are clinically significantly aberrant. Moreover, predictive values of single neurobiological measures are low at best [45, 46]. Therefore, in the current study, we use the biopsychosocial model and integrate neurobiological and psychosocial measures to identify clinically relevant subgroups of detained juveniles associated with youth offending outcomes, rather than identifying the role of individual neurobiological factors. By using an advanced form of latent class analysis, namely latent class regression analysis (LCRA; as advocated in [47]), it is explored whether groups can be identified that differ with respect to various psychosocial and neurobiological characteristics, and their relationship with reoffending. Moreover, to test whether individual cases can be correctly assigned to the subgroups and reoffending risk, we used a prototype ‘tool’ for calculating class membership based on the LCRA model. To evaluate possible added value of neurobiological measures, a model including these measures will be compared to a model with solely psychosocial predictors.

## Method

### Participants

**Fig. 1:**
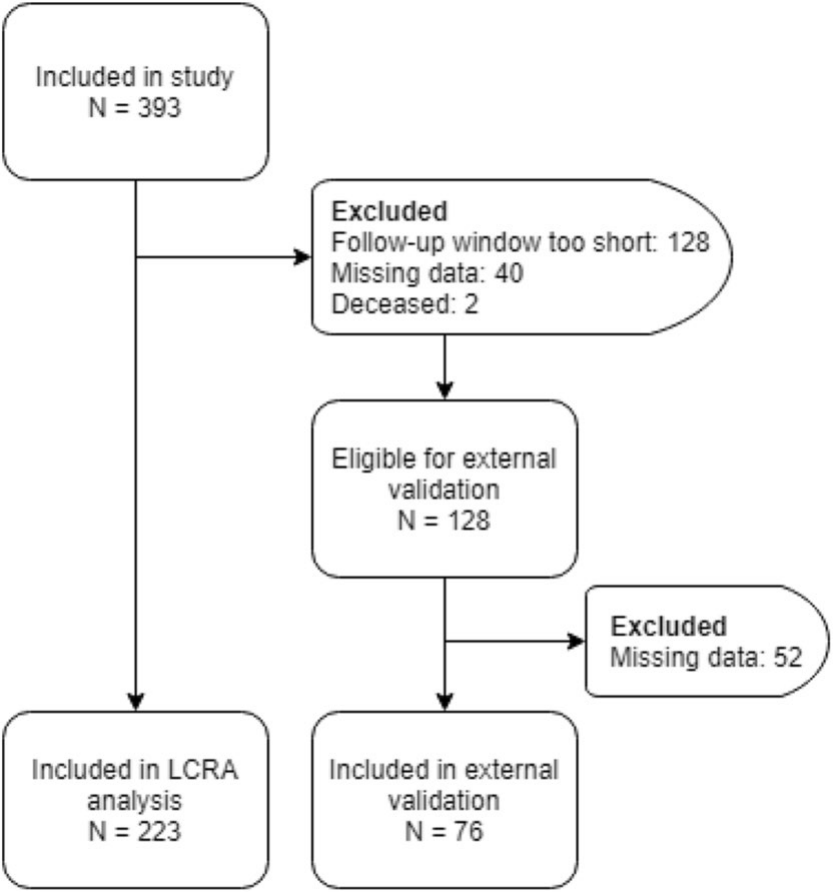
Flowchart of inclusion and exclusion

Participants were 393 detained male juveniles between the age of 12 and 24, the age range of adolescents that can be admitted within a Juvenile Justice Institution in the Netherlands,^1^ recruited from five juvenile justice institutions in the Netherlands. Exclusion criteria were unwillingness or inability to sign informed consent, insufficient command of the Dutch language, cardiac problems that would interfere with the heart rate measurements (e.g. arrhythmia and asthma), and inability to understand instructions and questionnaires, which was brought to our attention by institution staff. Recidivism rates within 12 months after release were obtained through judicial files. Upon follow-up, two participants were deceased, and for 128 participants the follow-up window was too short (< 18 months) to reliably determine recidivism. For this reason, these participants were excluded in the latent class regression analysis. Since we could not guarantee missingness completely at random we decided to exclude all participants with missing values on the neurobiological marker predictors (see Fig. 1 for a flowchart of inclusion and exclusion). The total sample for latent class regression analysis included 223 detained male juveniles (mean age = 18.49, *SD* = 1.73). The data of the participants with insufficient follow-up duration (n = 128), that were excluded from the latent class regression analysis, were used to evaluate the external validity of the model. For 52 of these 128 participants there were missing data, which allowed the model to be tested on 76 participants.

All participants, and when under the age of 18 also parents/caregivers, signed an informed consent document before participation. The study was approved by the Ethics Committee at the University of Amsterdam, and performed in accordance with the ethical standards described in the 1964 Declaration of Helsinki.

### Measures

Questionnaires concerning demographic characteristics provided information regarding age, non-Western minority status, socioeconomic status (SES), and presence of criminal peers. Non-Western ethnicity, as an indication of belonging to an ‘ethnic minority’, which has been related to recidivism risk [48, 49], was determined by the place of birth of parents (e.g. non-western, when one or both parents were born in a non-western country). In order to estimate neighborhood SES, participants were asked to provide the postal code of the address where they had lived the longest during their youth. This was then recoded into low, middle or high neighborhood SES based on data from Statistics Netherlands (CBS).^2^ To obtain an indication of the amount of criminal friends, participants were asked whether they have friends who are sometimes involved in crime (yes/no).

The Youth Psychopathic Traits Inventory-Short version (YPI-s; [50]) is a self-report measure to assess psychopathic-like traits in adolescents. It consists of three subscales measuring the interpersonal, affective and behavioral dimensions of psychopathic traits. The YPI-s consists of 18 items with a 4-point Likert scale ranging from 1 (does not apply at all) to 4 (applies very well). Higher YPI-s scores on any of the scales indicate a likelihood of a higher degree of psychopathic traits on that dimension. Research has supported the YPI three factor structure and has shown that the instrument is internally consistent [51]. This measure has been shown to identify detained youths with high levels of psychopathic-like traits [52].

The Brief Problem Monitor—Youth (BPM-Y; [53]) measures potential problems in youths’ functioning along three dimensions: Internalizing, Externalizing and Attentional Problems. The BPM is a rating instrument for monitoring children’s functioning and responses to interventions (RTIs). In the current study, only the youth self-report version of the BPM was used. The items are drawn from the Youth Self-Report (YSR) [54]. Each item is rated 0 (not true), 1 (somewhat true), or 2 (very true). Higher BPM-Y subscale scores are indicative of more problems along the relevant dimensions. Higher BPM-Y total scores are indicative of more overall problems. The BPM-Y was shown to have adequate test–retest reliability and validity, and showed good psychometric properties in a Norwegian sample of children and adolescents [53, 55].

The Adolescent Treatment Motivation Questionnaire (ATMQ) is a self-report instrument measuring treatment motivation. It was derived from the MTQ, based on the TTM of Prochaska and DiClemente [56]. The ATMQ consists of 11 items measuring the active phase of treatment motivation as a single construct, and is currently in use as part of the Dutch Routine Outcome Monitoring project in secure residential youth care. A 3-point answering scale with thumbs pictures is used for better comprehension. Items are rated untrue, somewhat true, or true. Higher scores on the scale for treatment motivation indicate greater treatment motivation. Internal consistency reliability was good (Cronbach’s alpha 0.84) for a sample of adolescents in Dutch secure juvenile facilities [57].

The short form of the Childhood Trauma Questionnaire (CTQ-SF; [58]) is a standardized, retrospective 28-item self-report inventory that measures the severity of different types of childhood trauma, producing five clinical subscales: Emotional Abuse, Physical Abuse, Sexual Abuse, Emotional Neglect and Physical Neglect. CTQ items are given a score of 1 through 5 on a five-point Likert type scale, ranging from 1 (never true), to 5 (very often true). Higher scores on the scales mean higher levels of whatever that scale represents. The CTQ is validated for adolescent psychiatric patients [59] and male and female street-youth [60]. The original CTQ consists of 70 items, but the version with 28 items is also validated for multiple populations [58]. Item 24 has been removed because experience and research showed a low validity of this item.

An indication of substance use frequency was obtained by means of self-reports of drug use. Participants were asked on an 11-item rating scale how many times they had used various drugs (e.g., alcohol, marijuana, ecstasy) in the last month and in their lifetime. Response choices increased in intervals of 10 (e.g. 1 = *never used*, 2 = *1–10 times,* 3 = *11–20 times*, with the last category being 11 = *91–100 times*). Many of these items were previously established with regard to predictive validity and reliability [61]. Data were recoded into the following categories: non-user, recreational user (one to 10 times in a year), multiple user of one substance (> 10 times in a year) with or without other recreational use, and multiple multidrug user (> 10 times in a year).

ANS-activity was measured using the VU- Ambulatory Monitoring System (AMS; [62]). Data were analyzed with VU-AMS software and data analysis support was offered by the VU-AMS department of VU University. Three electrodes were placed on the chest to measure participant’s electrocardiography (ECG), and four additional electrodes were placed on chest and back for assessment of impedance cardiography (ICG). Electrode placement was done according to the VU-AMS manual (http://www.vu-ams.nl/support/instruction-manual/). Baseline functioning was measured during a 5-min rest protocol (aquatic video, Coral Sea Dreaming, Small World Music Inc.; conform [63]). Reactivity was measured during two emotional film clips ('Mohamed', [64], 'The Champ', [65]), which were counterbalanced. This was done to prevent any effect of the order in which the clips were presented, for instance, due to loss of concentration over time. Preceding each clip, participants viewed one minute excerpts of the aquatic video while baseline functioning was measured. Values for both film clips and baselines were averaged, then change scores were created subtracting baseline averages from selected target episodes of the film clips. This resulted in rest as well as reactivity measures for heart rate (HR), and measures of sympathetic (pre-ejection period/PEP) and parasympathetic (respiratory sinus arrhythmia/RSA) branches of the ANS. The emotion evocation task and data preparations are described in more detail elsewhere [66].

Saliva (at least 0.1 ml) for cortisol and testosterone assessment was collected using a Salivette® (Sarstedt, Nümbrecht, Germany). All saliva samples were obtained on weekdays, between 12:00 and 18:00. A day before, as well as the hour before sampling, all participants were reminded of the sampling procedure. Additionally, they were reminded not to eat, drink (with the exception of water), smoke or brush their teeth during the hour before the start of the appointment. Ten minutes before sampling, participants were asked to rinse their mouth with tap water. A member of the research team gave verbal instructions before and during sampling. After saliva collection, a member of the research team wrote down subject number, date and exact sampling time. All samples were stored in the freezer the same day. Uncentrifuged samples were stored at -20 °C until analysis. Analyses were performed at the Endocrinology Laboratories of the University Medical Centre Utrecht.

Cortisol in saliva was measured without extraction using an in-house competitive radio-immunoassay employing a polyclonal anti-cortisol-antibody (K7348). [1,2-^3^H(N)]-Hydrocortisone (NET396250UC, PerkinElmer) was used as a tracer. The lower limit of detection was 1.0 nmol/L and inter-assay variation was < 7% at 3.3—30 nmol/L (n = 80). Intra-assay variation was < 4% (n = 10).

Testosterone in saliva was measured in duplicate using an in-house competitive radio-immunoassay employing a polyclonal anti-testosterone-antibody (Dr. Pratt AZG 3290). [1,2,6,7-^3^H]-Testosterone (NET370250UC, PerkinElmer) was used as a tracer following chromatographic verification of its purity. The lower limit of detection was 10 pmol/L. Inter-assay variation was 9.1, 4.3 and 5.6% at 95, 200 and 440 pmol/L respectively (n = 12, LKCH SL protocol 1610). Intra-assay variation was 7.2–2.5% at 38–92 pmol/L respectively (n = 10).

### Recidivism

Recidivism data were obtained from official records in the Judicial Documentation register of the Dutch Ministry of Justice, retrieved on July 7th 2017. New offenses were classified as general offenses, and further subdivided into violent and non-violent offenses. General recidivism was defined as any incident (including violent offenses) that led to an official judicial conviction, excluding technical breaches of order. Violence was defined as any (attempted) act intended to cause physical or psychological harm to others. Recidivism data were coded for the 12 months after release.

As there is always some delay between the incident and the registration in the official records, the Dutch Scientific Research and Documentation center advises a data-collection timeframe of 24 months for a follow-up period of 12 months, in order to ensure that criminal cases are fully closed and registered on the criminal records. When a shorter timeframe is used, it is more likely that committed offenses have not yet been registered. In the current study an 18 month timeframe was used, which may result in somewhat less certainty regarding the reconviction data collected for the 12 month follow-up time.

### Procedure

Juveniles were assessed individually in a test room inside the institution. Research staff were trained with regard to electrode placement and procedures of the tasks, and remained in the room for the entire period of testing. They followed a detailed written protocol including verbal instructions. Electrodes were placed on the juveniles’ chest, back, and fingers, and then connected to the VU-AMS device. Participants were instructed to sit still, and asked not to touch the electrodes. During the next ten minutes participants were asked to complete questionnaires on the computer to allow them to acclimate to the setting. During this period ANS parameters were measured as a natural baseline (acclimation period). After that, HR, HRV/RSA, and PEP were measured while juveniles completed tasks on the computer. These tasks consisted of a rest measure during a 5-min resting protocol, a countdown task (conform [67]) and the viewing of two film clips, interspersed with 1 min baselines. In case of having problems with sitting still, a gentle reminder was provided. After completion of the ANS measurements, participants were disconnected from the VU-AMS device and asked to collect saliva in a plastic tube. Then they continued with questionnaires and tasks on the computer for the remainder of the session. The total session lasted approximately 90 min. The participants were compensated for their time with a €5 stipend.

### Statistical analyses

Descriptive analyses were performed, and differences between the subgroups were examined using SPSS (Statistical Package for the Social Sciences, version 22). Subgroups were constructed consisting of different psychosocial and neurobiological measures using latent class regression analysis (Latent GOLD 4.0; [68]). A latent class *regression* analysis (LCRA) was performed because:

1. The core research question is to predict recidivism by a set of neurobiological markers. From previous findings and theory, we expect, that prediction is likely to be moderated by combinations of all kinds of psychosocial and background variables. As consequence a moderation analysis would be appropriate. In fact, the latent class regression analysis is such a moderation analysis. Instead of performing separate univariate moderation analyses for all kinds psychosocial and background variables, the latent class regression analysis searches for latent classes characterized by combinations of psychosocial and background variables that optimally moderate the different relationships between the neurobiological markers and recidivism.
2. Using LCRA we can form classes with predictive value for reoffending in *a one-step* model. This differs from the classic LCA analyses, where you first form classes and then test whether they predict reoffending, with the risk that the classes are not related to reoffending, or do not differ in reoffending risk.
3. Using LCRA we can form classes based on psychosocial factors and incorporate neurobiological predictors within those classes in the same model. This has the advantage that you can take into account that the influence of neurobiological factors may differ within the psychosocial classes.
4. Based on the LCRA model a ‘tool’ for calculating class membership can be created (see below), which can be used for internal and external validation, but also for calculating re-offending risk for ‘new’ cases.

The latent class regression model is shown in Eq. 1 in which y is the dependent variable reoffending after 12 months (no offending, non-violent offending, violent offending). In the latent class regression analysis there are two kinds of indicators, z^c^ and z^p^. The covariate indicators, z^c^ are independent variables that are used to form the latent classes, x. These are the psychological and background variables (age [continuous], SES [categorical], ethnicity [categorical], psychopathic traits [continuous], problem behavior [continuous], criminal friends [categorical], substance use [categorical], treatment motivation [continuous], and trauma [continuous]). The predictor indicators, z^p^, are also independent variables which are the neurobiological predictors in the regression analysis (HR reactivity, PEP reactivity, RSA reactivity, cortisol and testosterone [all continuous]) to predict the dependent variable (y), recidivism*.*

$$f~\left( {\left. y \right|\user2{z}^{c} ,~\user2{z}^{p} } \right) = ~\mathop \sum \limits_{x} \pi ~\left( {\left. x \right|\user2{z}^{c} } \right)~f~\left( {\left. y \right|~x,~\user2{z}^{p} } \right)$$

Latent class regression models were fitted for one through three classes. We used the following relative fit measures to evaluate the fit of the models. (1) The Bayesian Information Criterion (BIC) [69], (2) the sample-size adjusted BIC (SABIC), (3) the Akaike Information Criterion (AIC) [70], (4) the corrected AIC with penalty factor 3 (AIC3), and (5) the consistent AIC (CAIC) [71]. These measures have in common that they take into account—though in different ways—the sample size and the number of parameters to evaluate the fit. See Tofighi and Enders [72] for a comparison of the different indices. The lower the value of the indices, the better the relative fit of the model. Lubke and Neale [73] found through a simulation study that the AIC and SABIC showed better performance than the BIC and CAIC indices in determining the correct mixture model.

Internal validation of the model was done by evaluating the classification fit measures. In addition, an external validation was performed to evaluate the functioning and stability of the model. This was done by predicting class membership and recidivism in 76 participants who were not included in the dataset that was used to estimate the model parameters, due to too short follow-up periods. The class membership as well as the predicted reoffending category (no, non-violent, violent) was calculated using the research prototype tool provided on the website https://architecta.shinyapps.io/PredictingYouthReoffending/. The predicted reoffending category was compared to the observed reoffending category. Sensitivity, specificity, positive predictive value, and negative predictive value were calculated for the reoffending categories. This should be considered as a first step in external validation, as the follow-up window is relatively short and widely varies between the 76 participants. Finally, we analyzed whether the model with neurobiological markers can offer better prediction compared to a model with only psychosocial predictors, thus without neurobiological markers. Again, sensitivity, specificity, positive predictive value, and negative predictive value were calculated for the reoffending categories without neurobiological markers.

## Results

Table 1 shows five information criteria measures which evaluate the relative fit of the model and penalize for the number of parameters.^3^ The lower these fit measures, the better the model in terms of relative fit and parsimony. Unfortunately, although a very common result for latent class models, the different information criteria lead to a different number of classes. The BIC and CAIC would result in the 1 class model, while the AIC, AIC3 and the SABIC lead to 3 classes. Bootstrapped loglikelihood difference tests showed that the fit of the two-class model was better than the fit of the one-class model and the fit of the three class model was better than that of the two-class model. In the two class model, the fit statistics show that local independence could not be assumed. The L^2^ of the three class model was not significant, indicating that local independence could assumed. The classification errors (CE) show that the percentage of classification errors is very low, 2% for the three class model, and the interpretation of this model shows that the 3 class solution could well be interpreted. The class sizes were substantial for all three classes (0.62, 0.21 and 0.17 respectively). It was therefore decided to choose the three class model (see also Table 2).

**Table 1:** Information criteria of the model fit for the one, two and three class models resulting from latent class regression analysis for categories of offending within 12 months after detention

	Npar	L^2^	BIC(L^2^)	AIC(L^2^)	AIC3(L^2^)	CAIC(L^2^)	SABIC(L^2^)	df	*p*	CE	R^2^
1 class	18	383.92	− 724.55	− 26.08	− 231.08	− 929.55	− 7.49E+01	205	5.30E−13	0	0.03
2 class	54	268.71	− 645.10	− 69.29	− 238.29	− 814.10	− 1.10E+02	169	1.60E−06	0.01	0.38
3 class	90	154.03	− 565.13	− 111.97	− 244.97	− 698.13	− 143.63	133	0.1	0.02	0.73

BIC = Bayesian Information Criterion, SABIC = Sample-size Adjusted BIC, AIC = Akaike Information Criterion, AIC3 = corrected AIC with penalty factor 3, CAIC = Consistent AIC

Comparison class 1 vs class 2: -2LLDiff = 115.21, p < .01, Comparison class 2 vs class 3: -2LLDiff = 117.04, p < .001

**Table 2:** Descriptive statistics (percentages of categorical variables, means and standard deviations of continuous variables) for the overall sample and the three subgroups

	‘Low risk – psychopathic traits’ (N = 37) class 3		‘Medium risk—adverse environment’ (N = 141) class 1		‘High risk—externalizing’ (N = 45) class 2		Overall sample (N = 223)	
	%		%		%		%	
Etnicity								
Dutch	27.7		22.2		48.6		30	
Western	3.5		2.2		5.4		3.6	
Non-western	68.8		75.6		45.9		66.4	
Socioeconomic status								
Low	31.2		33.3		10.8		28.3	
Middle	66.7		64.4		67.6		66.4	
High	2.1		2.2		21.6		5.4	
Criminal friends								
Yes	71.6		88.9		56.8		72.6	
No	28.4		11.1		43.2		27.4	
Substance use								
Non-user	14.2		0.0		21.6		12.6	
Recreational user	18.4		17.8		0.0		15.2	
Multiple user 1 substance	28.4		73.3		40.5		39.5	
Multiple user > 1 substance	39.0		8.9		37.8		32.7	

	*M*	*SD*	*M*	*SD*	*M*	*SD*	*M*	*SD*
Age	18.66	1.70	18.13	1.38	18.29	2.13	18.49	1.73
PT Interpersonal dimension	10.11	3.76	10.40	2.72	12.76	3.95	10.61	3.72
PT Affective dimension	10.36	3.18	8.93	2.53	12.16	3.70	10.37	3.29
PT Behavior dimension	12.52	3.44	11.22	2.91	12.54	3.40	12.26	3.36
Internalizing problems	1.27	1.60	0.96	1.54	1.35	1.93	1.22	1.64
Externalizing problems	2.03	1.95	2.82	2.08	4.03	2.65	2.52	2.22
Attention problems	3.45	2.40	3.38	2.44	3.14	1.99	3.39	2.33
Treatment motivation	26.77	6.79	26.82	6.33	21.05	4.47	25.83	6.70
Trauma	39.04	7.45	43.71	14.05	48.54	15.46	41.56	11.23
HR rest	70.85	9.44	74.38	12.63	71.36	11.62	71.65	10.57
PEP rest	97.57	19.62	97.35	19.14	101.22	20.66	98.13	19.66
Lg RSA rest	1.85	0.24	1.81	0.27	1.85	0.19	1.84	.235
HR reactivity	− 2.93	2.40	− 3.41	2.80	− 2.64	2.92	− 2.98	2.57
PEP reactivity	0.41	2.75	− 0.16	2.52	− 0.39	3.59	.162	2.87
RSA reactivity	− 5.44	21.63	3.13	18.87	− 9.92	22.46	− 4.46	21.55
Cortisol	9.94	3.57	9.73	2.47	8.82	2.69	9.71	3.25
Testosterone	289.65	77.08	271.56	63.22	281.03	80.14	284.57	75.06

*PT* psychopathic traits, *HR* heart rate, *PEP* pre-ejection period, *Lg* transformed logarithmically, *RSA* respiratory sinus arrhythmia

**Fig. 2:**
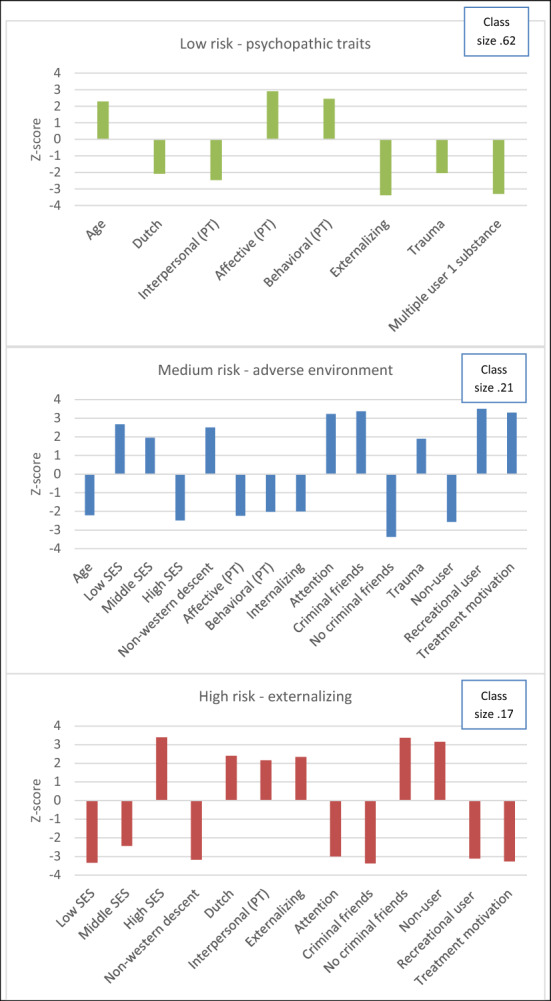
Subgroups of juvenile offenders (Z-scores > 1.80) with different relationships to the categories of reoffending behavior (no offending, non-violent offending and violent offending) within 12 months after release. PT = psychopathic traits

NB: The descriptives in Table 2 show the mean differences in the sample. Due to sample fluctuations these differences may not be reliable for the population. In order to interpret relevant differences at population level we focused on the differences that had a standardized score of 1.8 (see Fig. 2)

Descriptive statistics for the different classes are displayed in Table 2. An overview with descriptive statistics of neurobiological variables per recidivism type (no, non-violent, violent) is presented in Additional file 1: Table B in the addendum. Because the latent class regression analysis is an exploratory analysis and our aim is mainly to describe relevant patterns in the data we decided to be a bit more lenient with respect to significance levels by using a type one error rate of 0.07 instead of the commonly used 0.05. In order to interpret the classes we evaluated the effect of the covariates that predicted class membership. Figure 2 gives a graphic display of the three classes (see also Additional file 1: Table A in addendum for an overview of the effect of psychosocial covariates). All comparisons in the following section concern comparisons between the subgroups based on the Z-scores form the LCRA analyses.

In the *‘Low risk—psychopathic traits’ class*, the adolescents were on average older and were not often of Dutch descent. They had higher scores on the affective and behavioral dimensions of psychopathic traits (YPI-s). The juveniles scored lower on the interpersonal dimension of psychopathic traits (YPI-s), externalizing problems (BPM-Y), and trauma (CTQ-s). Few people reported multiple use of a substance. The probability of not reoffending within 12 months was 0.81, and the probability of reoffending was 0.14 for non-violent and 0.05 for violent reoffending. The ‘Low risk—psychopathic traits’ subgroup had a class size proportion (${\pi }_{x}$) of 0.62. This means that 62% of the participants in the sample would be assigned to this class, or, generalized to the population, that 62% of the population would be assigned to this class.

In the *‘Medium risk*—*adverse environment’ class*, the adolescents were on average younger and of non-western descent. They more often had a low or middle SES and more often reported having criminal friends. Individuals in this group scored low on the affective and behavior dimension of psychopathic traits (YPI-s) and internalizing problems (BPM-y). On average juveniles scored higher on attention problems (BPM-y) and trauma (CTQ-s). Individuals had a higher probability of being a recreational user and scored higher on treatment motivation. The probability of not reoffending was 0.45, with a probability of 0.31 for non-violent and 0.24 for violent reoffending. The ‘Medium risk—adverse environment’ subgroup had a class size proportion of 0.21.

Juveniles in the *‘High risk*—*externalizing’ class* on average had a high SES, were of Dutch descent and more often reported not having criminal friends. Individuals scored higher on the interpersonal dimension of psychopathic traits (YPI-s) and externalizing problems (BPM-y). They more often reported having few attention problems (BPM-y), and had a higher probability of being a non-user. Furthermore, they scored lower on treatment motivation. The probability of not reoffending was 0.27, with a probability of 0.43 for non-violent and 0.30 for violent reoffending. The ‘High risk—externalizing’ subgroup had a class size proportion of 0.17.

### Neurobiological predictors

**Fig. 3:**
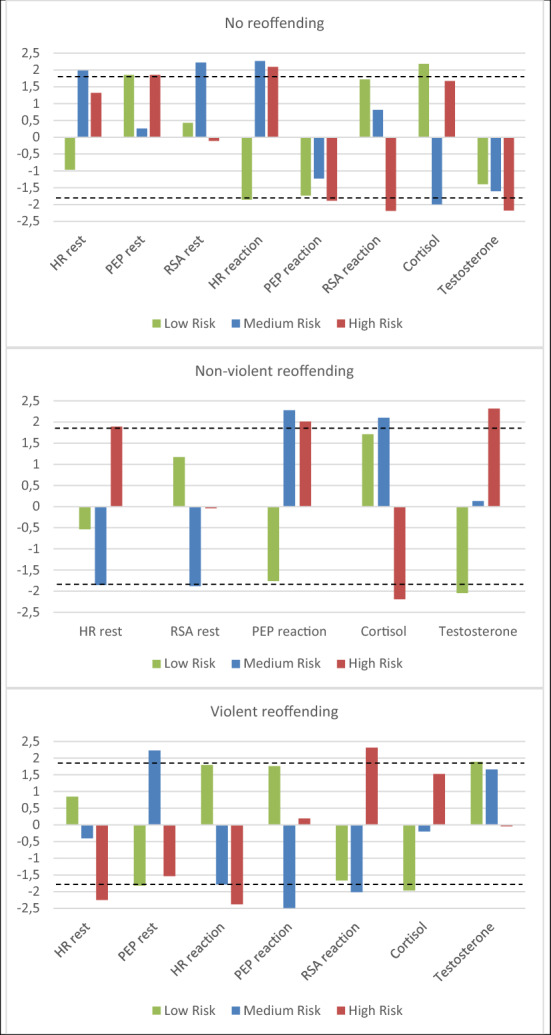
Neurobiological profiles for the three subgroups of juvenile offenders with different categories of reoffending (no offending, non-violent offending and violent offending) within 12 months after release

Furthermore, we interpreted the regression weights of the neurobiological predictors within the classes. Descriptive statistics for the neurobiological parameters in the different classes are displayed in Table 2. Figure 3 gives a graphic display of the three classes (see also Additional file 1: Table B in addendum for an overview of the effect of neurobiological predictors).

In *the ‘Low risk*—*psychopathic traits’ class*, there was a negative regression weight of HR reactivity (Z = − 1.86), and positive regression weights for cortisol (Z = 2.18) and PEP in rest (Z = 1.85), indicating that a lower HR reactivity and higher cortisol and PEP in rest were accompanied by a higher probability of not reoffending within 12 months. Lower testosterone (Z = − 2.05) was accompanied by a higher probability of non-violent reoffending. Lower PEP in rest (Z = − 1.82) and cortisol level (Z = − 1.96), and higher testosterone level (Z = 1.89), was accompanied by a higher probability of violent reoffending.

In the *‘Medium risk*—*adverse environment’ class*, higher HR and RSA in rest (Z = 1.98 and 2.22 respectively), higher HR reactivity (Z = 2.26), and lower cortisol level (Z = − 1.99) were accompanied by a higher probability of not reoffending. For non-violent reoffending, lower HR and RSA in rest (Z = 1.86 and -1.88 respectively), and higher PEP reactivity and cortisol level (Z = 2.27 and 2.10 respectively) were accompanied with a higher probability of non-violent reoffending. Lower PEP and RSA reactivity (Z = − 2.49 and − 2.01 respectively) and higher PEP in rest (Z = 2.23) were associated with a higher probability of violent reoffending.

Finally, in the *‘High risk*—*externalizing’ class*, a higher PEP in rest (Z = 1.85) and HR reactivity (Z = 2.09), and a lower PEP reactivity (Z = − 1.88), RSA reactivity (Z = − 2.19), and testosterone level (Z = − 2.17) were accompanied by a higher probability of not reoffending. For non-violent reoffending, a higher HR in rest (Z = 1.89), PEP reactivity (Z = 2.01), and testosterone level (Z = 2.32), and a lower cortisol level (Z = − 2.19) were accompanied by a higher probability of non-violent reoffending. Lower HR in rest (Z = − 2.25) and HR reactivity (Z = − 2.38), and higher RSA reactivity (Z = 2.31) were accompanied by a higher probability of violent reoffending.

### External validation of the latent class regression model

The data of the 76 participants with insufficient follow-up duration for the LCRA analysis was used as a reference sample to evaluate the external validity of the latent class regression model. The class membership as well as the predicted reoffending category (no, non-violent, violent) was calculated using the research tool provided on the website https://architecta.shinyapps.io/PredictingYouthReoffending/. The predicted reoffending category was compared to the observed reoffending category. The results of this analysis are presented in Table 3. For 92.1% (n = 70) of the participants, the assignment to a class and prediction of reoffending was correct. Sensitivity, specificity, positive predictive value (PPV), and negative predictive value (NPV) were calculated for the reoffending categories (see Table 4).

**Table 3:** Observed and predicted counts of reoffending (no, non-violent, violent) for participants with insufficient follow-up duration* with and without neurobiological predictors

Observed reoffending	Including neurobiological predictors			Without neurobiological predictors			Total
	Predicted reoffending			Predicted reoffending			
	No	Non-violent	Violent	No	Non-violent	Violent	
No	51	1	1	0	37	16	53
Non-violent	4	13		0	2	15	17
Violent			6	0	1	5	6
Total	55	14	7	0	40	36	76

^*^ Considering the short follow-up duration, these data should be interpreted with caution

**Table 4:** Probabilities of correct prediction of reoffending type (sensitivity, specificity, positive predictive value (PPV), and negative predictive value (NPV)) with and without neurobiological predictors

	Reoffending					
	Including neurobiological predictors			Without neurobiological predictors		
	No	Non-violent	Violent	No	Non-violent	Violent
Sensitivity	0.93	0.93	0.86	0.00	0.09	0.10
Specificity	0.90	0.94	1.00	0.30	0.58	0.98
PPV	0.96	0.76	1.00	0.00	0.12	0.83
NPV	0.83	0.98	0.99	1.00	0.36	0.56

Finally, to assess whether the model with neurobiological markers can offer better prediction compared to a model with only psychosocial predictors, sensitivity, specificity, PPV, and NPV were calculated for the reoffending categories without neurobiological markers in the model. The results show that when neurobiological predictors are omitted, predictions based on the model with only sociological and psychological data deteriorate (see Tables 3 and 4).

## Discussion

The present study aimed to use a biopsychosocial model to identify clinically relevant subgroups of juvenile offenders through latent class regression analysis. The motivation for this venture was to test whether this approach is useful to develop an integrated model of neurobiological and psychosocial risk factors to improve predictive models of reoffending, as well as to equip clinicians within juvenile justice institutions with a method to assign individuals to relevant subgroups for reoffending risk, which could subsequently inform intervention. Based on psychosocial and neurobiological characteristics of 223 juvenile offenders, three relevant subgroups in relation to reoffending behavior were identified: a ‘low risk—psychopathic traits’ subgroup, a ‘medium risk—adverse environment’ subgroup, and a ‘high risk—externalizing’ subgroup, for which relationships between neurobiological factors and type of reoffending differed. Both psychological, social and neurobiological factors contributed to the distinction between these subgroups. The three class model could accurately predict the latent class of juveniles, both internally for the targeted population (98%), as well as externally for new juveniles (92%). The model showed good accuracy in terms of sensitivity, specificity, and positive and negative predictive value. Predictions deteriorated when a model with solely social and psychological factors was used, removing neurobiological predictors from the model, demonstrating added value of neurobiological measures.

Psychosocial offender characteristics that proved to be important in the current study, such as previous offending behavior, problem behavior, psychopathic traits, and substance use, have been shown to be significant for allocation to homogenous subgroups of delinquent adolescents in earlier research as well [2, 6, 9–12, 15]. Individuals in the ‘Low risk—psychopathic traits’ subgroup were characterized by the least risk factors. They were somewhat older, showed higher affective and behavioral psychopathic traits, but less externalizing behavior. The finding of psychopathic traits in the low risk group is in contrast with most studies showing high risks of recidivism in adolescents with psychopathic traits (for a meta- analysis: [74]. However, it should also be noted that the high risk group actually scored highest on psychopathic traits. The main difference between the low risk and high risk group is that the low risk group shows psychopathic traits, but relatively low externalizing problems, while the high risk groups scored high on both. Apparently, this is a fundamental difference which may be important in the interpretation of the high psychopathy scores on the questionnaires, and the subsequent risk of reoffending. Similar subgroups *of high psychopathic traits and high psychopathic traits plus externalizing problems* and subsequent risks for delinquent behavior have previously been found in children as well [75]. Individuals in the ‘Medium risk—adverse environment’ subgroup more often presented trauma and attentional problems. This subgroup appears to be characterized by poorer environmental conditions: being more likely to come from a disadvantageous neighborhood, have criminal friends, and use substances recreationally. Individuals in the ‘High risk—externalizing’ subgroup were characterized by relatively positive environmental circumstances, but high externalizing problems and interpersonal psychopathic traits. This is in concordance with previous studies where individual factors are more strongly related to recidivism as opposed to only environmental mental health factors. Such findings are explained by the “social push” hypothesis [23] which argues that where an antisocial child lacks *social* factors that *push* or predispose them to antisocial behavior, then neurobiological risk factors more likely explain antisocial behavior. However, it contradicts studies where the combination of environmental and individual mental health factors usually leads to highest risk of reoffending [13, 76].

By using the LCRA approach the neurobiological parameters initially contributed to the formation of the three classes. Although the neurobiological differences between the subgroups may not seem to have any clinical relevance in isolation, they do have a distinctive value within the prediction model. Moreover, by incorporating the neurobiological predictors within those classes, it was shown that they further differentiated within the classes which individual is at risk of no, non-violent or violent recidivism based on specific neurobiological profiles. It is important to note that with this approach, the model assesses the differential effects of neurobiological factors within each subgroup, which means that differences in neurobiological measures do not directly mean that the values reflect a high or low value of the predictor under consideration, but only refer to differences in neurobiological profiles between subgroups. This is fundamentally different from assessing neurobiological differences between delinquent juveniles and controls as was done in most previous studies, and therefore also shows a more complex picture of relationships between neurobiological measures and reoffending as compared to earlier research. This may also explain why in previous studies where neurobiological factors were related to reoffending in the total group of juvenile offenders show small and inconsistent results (De Vries-Bouw et al. [35]). Moreover, it also shows why there is currently no consensus on when biomarker levels are clinically significantly aberrant and development of cut-off values for neurobiological factors has so far been unsuccessful. This is where a research tool as discussed within the current study may be important, as it is better equipped than e.g. the clinical view at differentiating small differences within heterogeneous groups of already delinquent juveniles. This study underlines that the importance lies not in establishing cut-offs, but in determining which variables provide the most information, especially when trying to establish reoffending risk in groups of severely delinquent juveniles. The main advantage of the current approach is that, even though the model is quite complex, it can still be valuable for practical usage, because the tool can handle continuous values of neurobiological measures, which means there is no need for establishing strict cut-off values or clinical interpretation of neurobiological profiles.

However, some neurobiological factors emerge more consistently than others. Several of the relationships found in the current study correspond to relationships between neurobiological measures and reoffending from earlier research. For both groups with higher (violent) reoffending risk for example, parasympathetic nervous system (PNS; or RSA) reactivity comes forward as an important factor for violent reoffending. Within the “High risk—externalizing” subgroup it was a somewhat higher, and within the “Medium risk—adverse environment” subgroup lower PNS reactivity that was associated with violent reoffending. Aberrant PNS reactivity was previously found to be predictive of reoffending [77, 78]. Furthermore, HR weighed in the allocation to risk subgroups. The finding that a lower HR, both at rest and reactivity, is associated with (violent) reoffending (in the “High risk—externalizing” subgroup) is also conform previous findings [31–33, 77]. In more serious risk groups, these two biological markers, HR and PNS (re)activity, therefore seem relevant. They appear to be in the forefront among juveniles who commit (serious) recidivism. In the current study, inclusion of neurobiological measures predicted models of reoffending better than the use of solely psychological and social factors. This provides further support for the belief that conducting research from a biopsychosocial perspective can be fruitful. From previous literature, as stated, neurobiological measures in isolation appear to have moderate value in predicting subsequent behavior. However, when these measures are examined in concert with other psychosocial factors, this can significantly increase the predictive value (see also [46, 79, 80]).

### Study limitations

It is important to note that the described model should be considered as a preliminary model in a specific subgroup of delinquent juveniles. First, the current model only applies to the group of delinquent juveniles from juvenile justice institutions that are already severely delinquent and at high risk of reoffending. It cannot as a matter of course be argued that the links between the factors determining the subgroups and reoffending can be generalized to risk factors for delinquent behavior in the general population. However, the results can support existing ideas and theories on risk taxation for (violent) reoffending. The current study using LCRA analyses resulted in finding informative subgroups with different risks of reoffending [46, 79]. Within juvenile justice institutions, assessment of the risk for reoffending is an important issue, and this model, although it should be replicated in a separate sample, provides a first step to improve current risk assessment by including neurobiological factors as well. This is a first attempt to test whether the inclusion of neurobiological factors can be used for predicting recidivism. However, longitudinal studies including repeated assessments of both psychosocial and neurobiological factors are needed to make any claims on change in risk during the stay in the institution and how this relates to specific interventions within the institutions and subsequent recidivism risks. Second, it was not possible to enter all characteristics that were collected under the current project in the analyses in view of statistical power. Moreover, it is likely that other potentially important information that we are not yet aware of was not collected in the current study. Furthermore, the ratio of sample size to number of parameters did not allow to examine the fit of models with more than three classes. Adding other information could possibly change the composition of the subgroups. Third, it is important to underline that questionnaires in this study were self-report measures. For future studies, a combination of information from different sources may be desirable (e.g. a combination of self-report and other report). Should juveniles in clinical practice prove to be reluctant to provide information, information from other sources may offer a helpful solution. Finally, a follow-up period of 12 months for recidivism data is relatively short to allow for firm statements. Therefore, follow-up studies with a longer follow-up duration are necessary. If the follow-up duration is relatively short, there is a possibility that individuals will be acquitted (on appeal), or that offences that were committed were in fact not yet processed at the time of the recidivism request.

### Implications for clinical practice

The current study offers a first attempt to provide a new approach to increase the insight into the use of biopsychosocial profiles of juvenile offenders and reoffending in clinical forensic practice. The approach appears useful to develop an integrated model of neurobiological and psychosocial risk factors to improve predictive models of reoffending, as well as to equip clinical practice with a method to assign individuals to relevant subgroups for reoffending risk, which could subsequently inform intervention. At this stage, the main value of this study lies in the fact that we have shown that the LCRA technique may help us in using neurobiological factors to better predict who is at high risk of recidivism and who is not. This may inform professionals about which adolescents are most in need of (intensive) treatment. This seems a promising first step for integrating neurobiological risk factors in clinical practice. To facilitate practical use, a research tool prototype was constructed for the current study that could be developed into a clinical tool for professionals in the future. The research tool prototype correctly classified a large proportion of juveniles to one of the distinguished subgroups, showing both good internal and external validity of the three subgroups. At the moment, however, the research tool is still a preliminary model in a relatively small sample of juvenile delinquents and should be further developed and validated. Further research and replication of the findings are necessary, at the least with a larger sample, before the tool can be used to inform intervention of young offenders. Furthermore, at present the current prototype is not capable of dealing with missing information; a tool that is capable of this is under development. Future studies will have to reveal whether such a tool can be helpful in clinical practice.

## Supplementary Information

**Additional file 1: Table A.** Overview of Z-scores for 3 class model for three categories of recidivism (no offending/ non-violent offending/ violent offending) within 12 months after detention. **Table B.** Mean scores and standard deviations for neurobiological measures for the three subgroups for the categories of reoffending (no, non-violent and violent).

## Data Availability

The datasets used and/or analysed during the current study are available from the corresponding author on reasonable request.

## Abbreviations

ANS: Autonomic nervous system
HR: Heart rate
PNS: Parasympathetic nervous system
HRV: Heart rate variability
RSA: Respiratory sinus arrhythmia
SNS: Sympathetic nervous system
PEP: Pre-ejection period
ECG: Electrocardiography
ICG: Impedance cardiography
VU-AMS: VU-Ambulatory Monitoring System
SES: Socio-economic status
YPI-s: Youth Psychopathic Traits Inventory-Short version
PT: Psychopathic traits
BPM-Y: Brief Problem Monitor—Youth
ATMQ: Adolescent Treatment Motivation Questionnaire
CTQ-SF: Childhood Trauma Questionnaire—short form
LCRA: Latent class regression analysis
PPV: Positive predictive value
NPV: Negative predictive value

## Footnotes

1 In the Netherlands, juvenile justice ends at age 18. However, the recent adolescent criminal law, which has been in place since 2014, states that, based on developmental status, juvenile justice may be applied until the age of 23, with a maximum penalty of 2 years.

2 In the Netherlands, postal codes consist of a number of connected streets that generally attract the same type of households. Neighborhood income, which was provided by the Central Bureau of Statistics (CBS), was used to determine neighborhood SES.

3 Because of sparse frequency tables the *p*-value associated with the Chi square statistic cannot be trusted. Therefore, relative fit measures were used instead of absolute fit measures.
