# Supplementary material for: Using the biopsychosocial model for identifying subgroups of detained juveniles at different risk of re-offending in practice: a latent class regression analysis approach
Source: Child Adolesc Psychiatry Ment Health. 2021 Jun 22;15:33. doi: 10.1186/s13034-021-00379-1 (PMC8218478; doi:10.1186/s13034-021-00379-1)
Supplement: Supplementary file 1 — Additional file 1: Table A. Overview of Z-scores for 3 class model for three categories of recidivism (no offending/ non-violent offending/ violent offending) within 12 months after detention. Table B. Mean scores and standard deviations for neurobiological measures for the three subgroups for the categories of reoffending (no, non-violent and violent). [file 13034_2021_379_MOESM1_ESM.docx]

**Addendum**

|  | **Table A.** Overview of Z-scores for 3 class model for categories of recidivism (no offending/ non-violent offending/ violent offending) within 12 months after detention | | | | |
| --- | --- | --- | --- | --- | --- |
|  | |  | **Low Risk** | **Medium risk** | **High risk** |
| Class size | |  | 0.62 | 0.21 | 0.17 |
| No recidivism within 12m | |  | 0.81 | 0.45 | 0.27 |
| Non-violent recidivism within 12m | |  | 0.14 | 0.31 | 0.43 |
| Violent recidivism within 12m | |  | 0.05 | 0.24 | 0.30 |
|  | |  | **Z-scores** | | |
| Intercept | |  | -1.44 | -0.10 | 1.00 |
| Age | |  | 2.29* | -2.21* | 1.15 |
| Low SES | |  | 1.16 | 2.67* | -3.34* |
| Middle SES | |  | -0.31 | 1.95* | -2.43* |
| High SES | |  | -0.58 | -2.48* | 3.40* |
| Ethnicity: Non-western descent | |  | 0.29 | 2.50* | -3.18* |
| Ethnicity: Western descent | |  | 1.13 | -1.28 | 1.16 |
| Ethnicity: Dutch | |  | -2.08* | -1.20 | 2.41* |
| Psychopathic traits: Interpersonal dimension | |  | -2.46* | -1.52 | 2.17* |
| Psychopathic traits: Affective dimension | |  | 2.90* | -2.23* | 0.18 |
| Psychopathic traits: Behavior dimension | |  | 2.45* | -2.02* | 0.58 |
| Internalizing problems | |  | 1.58 | -2.00* | 0.73 |
| Externalizing problems | |  | -3.38* | 0.02 | 2.34* |
| Attention problems | |  | 1.00 | 3.23* | -2.99* |
| Report criminal friends | |  | 0.84 | 3.37* | -3.37* |
| Report no criminal friends | |  | -0.84 | -3.37* | 3.37* |
| Trauma | |  | -2.03* | 1.89* | -1.14 |
| Substance use: Non-user | |  | -0.16 | -2.57* | 3.16* |
| Substance use: Recreational user (1-10) | |  | 1.69 | 3.50* | -3.11* |
| Substance use: Multiple user 1 substance (>10) | |  | -3.30* | 0.58 | 1.69 |
| Substance use: Multiple multidrug user (>10) | |  | 0.16 | -1.65 | 1.78 |
| Treatment motivation | |  | 2.18 | 3.29* | -3.27* |
|  | |  |  |  |  |
|  | Neurobiological predictors | | | | |
| Intercept | | No offending | -1.46 | -1.72 | -0.91 |
|  | | Non-violent offending | -1.59 | 1.79 | -0.67 |
|  | | Violent offending | 1.55 | -1.44 | 1.31 |
| HR rest | | No offending | -0.97 | 1.98* | 1.32 |
|  | | Non-violent offending | -0.54 | -1.86* | 1.89* |
|  | | Violent offending | 0.84 | -0.40 | -2.23* |
| PEP rest | | No offending | 1.85* | 0.26 | 1.85* |
|  | | Non-violent offending | 1.77 | -1.68 | -1.25 |
|  | | Violent offending | -1.82* | 2.23* | -1.53 |
| RSA rest | | No offending | 0.43 | 2.22* | -0.11 |
|  | | Non-violent offending | 1.17 | -1.88* | -0.04 |
|  | | Violent offending | -0.84 | 0.18 | 0.12 |
| HR reaction | | No offending | -1.86* | 2.26* | 2.09* |
|  | | Non-violent offending | -1.71 | -1.77 | 0.74 |
|  | | Violent offending | 1.79 | -1.78 | -2.38* |
| PEP reaction | | No offending | -1.73 | -1.23 | -1.88* |
|  | | Non-violent offending | -1.76 | 2.28* | 2.01* |
|  | | Violent offending | 1.76 | -2.49* | 0.19 |
| RSA reaction | | No offending | 1.72 | 0.81 | -2.19* |
|  | | Non-violent offending | 1.49 | 0.95 | -1.71 |
|  | | Violent offending | -1.66 | -2.01* | 2.31* |
| Cortisol | | No offending | 2.18* | -1.99* | 1.67 |
|  | | Non-violent offending | 1.71 | 2.10* | -2.19* |
|  | | Violent offending | -1.96* | -0.19 | 1.52 |
| Testosterone | | No offending | -1.39 | -1.60 | -2.17* |
|  | | Non-violent offending | -2.05* | 0.13 | 2.32* |
|  | | Violent offending | 1.89* | 1.66 | -0.04 |
| *Z-score with a value >1.80 | | | | | |

| ***Table B.*** Mean scores and standard deviations for neurobiological measures for the three subgroups for the categories of reoffending (no, non-violent and violent) | | | | | | | | | |
| --- | --- | --- | --- | --- | --- | --- | --- | --- | --- |
|  | ***Low risk – psychopathic traits*** | | | ***Medium risk – adverse environment)*** | | | ***High risk – externalizing*** | | |
|  | *No*  *N = 68* | *Non-violent*  *N = 19* | *Violent*  *N = 6* | *No*  *N = 11* | *Non-violent*  *N = 9* | *Violent*  *N = 7* | *No*  *N = 3* | *Non-violent*  *N = 12* | *Violent*  *N = 8* |
|  | ***M (SD)*** | ***M (SD)*** | ***M (SD)*** | ***M (SD)*** | ***M (SD)*** | ***M (SD)*** | ***M (SD)*** | ***M (SD)*** | ***M (SD)*** |
| HR rest | 71.11 (10.24) | 71.63 (8.148) | 69.36 (8.54) | 72.70 (6.98) | 77.71 (19.77) | 77.22 (16.93) | 73.74 (9.00) | 71.82 (8.57) | 66.48 (9.56) |
| PEP rest | 99.09 (19.71) | 96.58 (21.75) | 93.55 (17.61) | 91.57 (19.07) | 103.73 (26.14) | 101.83 (21.52) | 121.28 (16.99) | 93.749 (17.22) | 91.53 (16.42) |
| Lg RSA0 rest | 1.81 (0.24) | 1.88 (0.19) | 1.96 (0.21) | 1.83 (0.14) | 1.73 (0.30) | 1.74 (0.26) | 1.90 (0.19) | 1.84 (0.21) | 1.92 (0.08) |
| HR reactivity | -3.08 (2.40) | -3.81 (2.82) | -2.94 (2.55) | -3.73 (2.33) | -4.29 (4.64) | -6.10 (4.16) | -2.63 (4.06) | -3.22 (2.97) | -4.00 (3.53) |
| PEP reactivity | 0.43 (3.03) | 0.75 (2.54) | 1.00 (3.23) | -0.13 (2.97) | -0.29 (2.43) | -1.41 (2.25) | -4.60 (4.39) | -0.25 (3.68) | -0.50 (4.91) |
| RSA reactivity | -6.87 (19.37) | -3.98 (16.53) | -14.93 (31.56) | -2.94 (11.65) | -2.62 (20.90) | -1.14 (25.17) | -14.49 (25.00) | -3.94 (13.99) | 6.46 (4.81) |
| Cortisol | 10.44 (3.77) | 8.64 (2.84) | 7.43 (3.07) | 8.36 (1.67) | 11.22 (2.68) | 10.50 (1.95) | 9.30 (1.06) | 8.52 (1.90) | 8.73 (3.50) |
| Testosterone | 289.57 (71.77) | 268.05 (90.30) | 294.17 (72.62) | 260.64 (52.53) | 269.89 (57.98) | 254.57 (46.94) | 267.00 (26.46) | 303.00 (88.78) | 289.50 (70.31) |
| *Note.* Lg = transformed logarithmically; RSA = respiratory sinus arrhythmia | | | | | | | | | |
